# Nutrition in cancer patients: analysis of the forum of women´s self-help association against cancer

**DOI:** 10.1186/s40795-025-01027-z

**Published:** 2025-02-13

**Authors:** A. Fettig, V. Mathies, J. Hübner

**Affiliations:** 1 Medical Doctor Universitätsklinikum Jena, Am Klinikum 1, 07747 Jena, Germany; 2 Klinik für Innere Medizin II, Universitätsklinikum Jena, Am Klinikum 1, 07747 Jena, Germany

**Keywords:** Cancer nutrition, Online support forums, Dietary changes, Patient support, Evidence-based nutrition

## Abstract

**Background:**

Online forums play a crucial role for cancer patients seeking nutrition-related information and support. This study investigated the most common nutrition-related questions and concerns among members of the Women’s Self-Help Association Against Cancer, focusing on emotional, physical, and practical aspects of dietary changes in cancer patients.

**Methods:**

A mixed-methods approach was used, combining qualitative and quantitative content analysis of 5314 forum responses. The themes identified included common questions, patient contributions, physician involvement, and the impact of the COVID-19 pandemic on the discussions.

**Results:**

In total, 2246 posts across 22 threads were analyzed, spanning 3867 days and receiving 654,100 visits. Key topics included 41 themes and 356 questions, with common inquiries like “Has your diet changed since your diagnosis?” (45 responses) and “Is sugar allowed in your diet?” (29 responses). There were 4958 contributions, with 558 evidence-based responses (e.g., recommending balanced diets) and 200 non-evidence-based responses (e.g., fasting, cancer-specific diets). Concerns regarding sugar (188 responses), dairy (121 responses), and emotional stress (187 responses) were common. Despite evidence-based recommendations from healthcare professionals supporting balanced diets, members frequently encounter non-evidence-based advice on fasting- and cancer-specific diets, leading to significant emotional and nutritional challenges. Additionally, Members emphasized the need to balance the enjoyment of food with dietary restrictions.

**Conclusion:**

The forum is a valuable resource for sharing experiences and advice; however, non-evidence-based content underscores the need for moderation and expert input. Collaboration between medical professionals and moderators can improve content reliability, enabling informed dietary decisions for cancer patients.

## Introduction

Food is an essential part of our everyday life, and it is important to understand how healthy nutrition is correlated with our health and overall well-being. Furthermore, nutrition is an important factor in patients with comorbidities such as cancer [2, 19]. Oncological diseases can arise in the course of interactions between genetic and environmental factors, including nutritional factors. Nutrition plays a crucial role in the treatment outcomes of cancer patients. (Faculty of Pharmacy Kampala International University Uganda & David [6]), Of course, no food alone can prevent tumors or heal cancer [3].

Living with cancer presents significant challenges, especially regarding to managing daily nutrition among patients with disease- and treatment-related side effects. Sharing these struggles with others who understand the journey can provide valuable insights and a sense of connection, turning shared experiences into sources of strength and support.

This self-help approach has evolved from local group meetings to robust local and online support organizations [7].

The Women’s Self-help Association against Cancer (Frauenselbsthilfe Krebs; FSH) is an organization that developed from a meeting of cancer-stricken women into a network with approximately 30,000 participants. It is a self-help group for cancer patients, highlighting the strong need for self-help in this population [4]. The forum is the oldest and one of the largest self-help networks for cancer patients in Germany [13]. According to the official website of the “Frauenselbsthilfe Krebs,” there are 8,156 topics with overall 518,800 responses and 7,628 active members.

Web-based self-management support has many advantages [1] but also disadvantages because false information can spread rapidly, and nutrition may differ for every individual patient [13]. This study was conducted to address the gap in evidence-based nutritional guidance for cancer patients within online forums, aiming to improve the quality of information shared, reduce misinformation, and provide accurate and reassuring support to elevate patient outcomes and quality of life.

In the present study, we focused on questions concerning nutrition in cancer, the nutritional habits of family members, online communication methods, evidence-based and non-evidence-based responses, and personal journeys. Our aim was to obtain an overview of the importance of nutrition and its relationship to nutrition, and to understand the significance of the role of nutrition in individuals’ overall personal health. This study also aimed to provide insights for doctors regarding areas where better education and information about nutrition in cancer patients are needed.

## Materials and methods

### Material

The Women’s Self-help Association welcomes female patients with various forms of cancer and male patients with breast cancer, with a majority of members having breast or gynecological cancer. In 2012, the digital forum was established, providing free access to all threads where users can create profiles and initiate or respond to discussions on various topics, with all user information and data being voluntarily posted (https://forum.frauenselbsthilfe.de). The forum is structured by patient volunteers who serve as moderators and adhere to rules that forbid false information, advertising, and offensive speech. On the main page of the official website of the Women’s Self-help Association, the topics are categorized under different categories.

In the present work, we focused on the titles that are listed under the main category “How can I help myself?”, especially by analyzing the participants’ responses on nutrition, malnutrition and other topics related to food.

With the permission of the Women’s Self-help Association, all threads within the forum,

spanning from January 6th, 2013, to September 27th, 2023, were subjected by A.F. to quantitative evaluation for nutrition in cancer patients.

We assessed threads from six sub-categories, which are listed under the main category “How can I help myself?”. We checked all six sub-categories for nutrition related topics. In four sub-categories “side-effect management”, “dealing with cancer”, “how can I deal with my anxious thoughts”, and “and more”, there were no posts concerning nutrition. In the remaining two sub-categories “Nutrition, Exercise and relaxation” and “Complementary treatment options” nutrition related posts could be analyzed.

A ‘thread’ is initiated by a guest or a member of the Women’s Self-help Association, who.

poses a question or chooses a topic for discussion with other members. Threads are used to organize sub-categories for members to know exactly where to chat about the problems or suggestions they have, which in turn promotes extended conversations on.

specific subjects. Members or guests can engage and establish conversations via multiple posts within threads.

Threads can also be used to explore and search for coping strategies to help them live with their diagnosis by reading all posts.

### Methods

#### Quantitative analysis

For our quantitative analysis, we focused on the title of the threads, their duration, the total.

number of posts, the average number of posts per day during the peak period and.

the total number of visits in all threads. In addition, we counted all responses concerning nutrition in the patients with cancer. There can be multiple responses in one post, which results in a wide variety of nutritional content in one thread. The content of these responses was then analyzed qualitatively. For the quantitative analysis and statistical analysis, we used Microsoft Excel.

#### Qualitative analysis

**Fig. 1:**
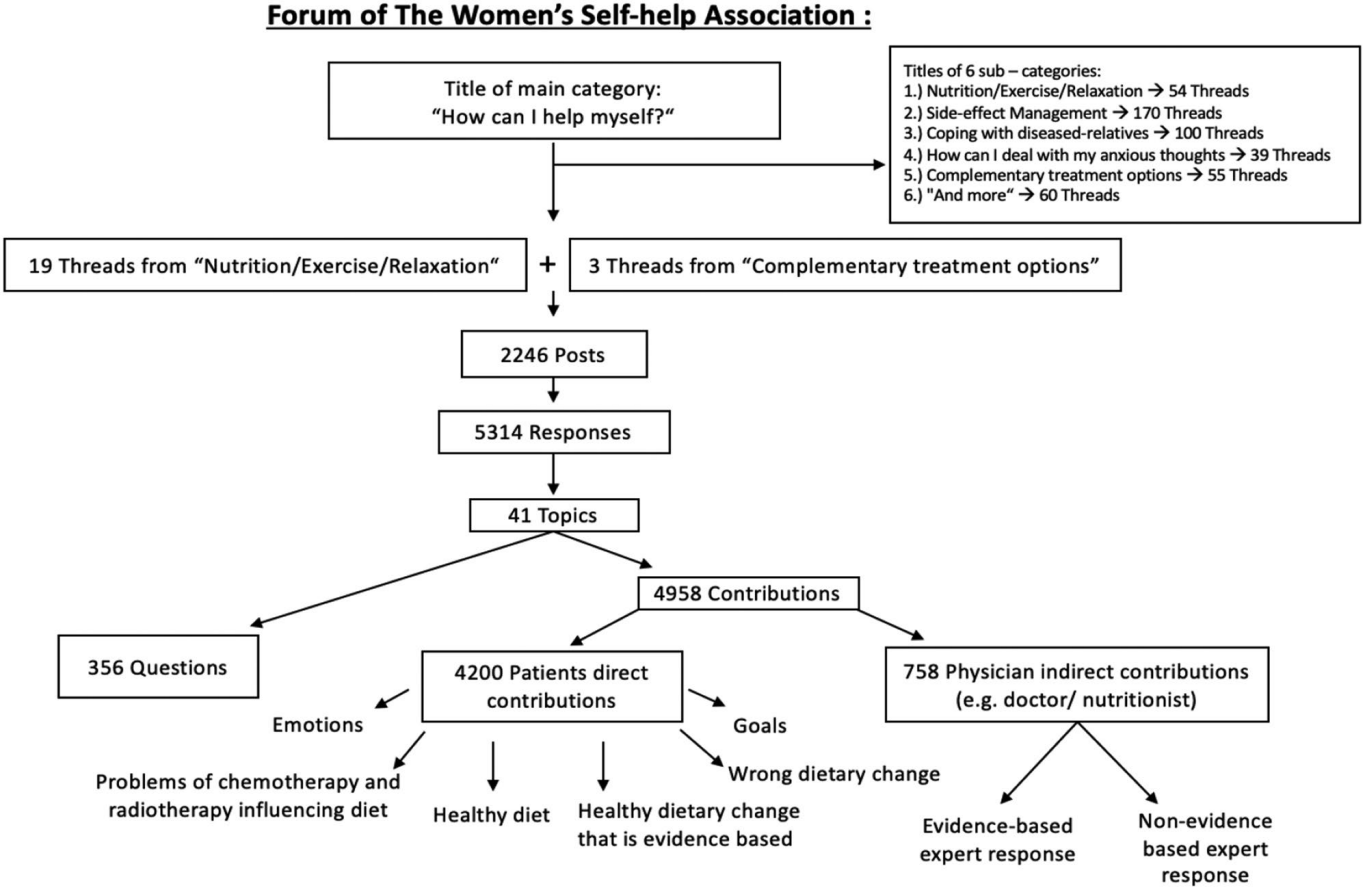
Qualitative analysis. In one thread, there are multiple posts and questions. Posts are made by members in all threads to establish a conversation. There can be multiple responses in one post. A topic is a result of different content in the response

A qualitative assessment was conducted, considering the content of each response and dividing it into different nutritional topics (Fig. 1). A topic includes everything that a member talked about in each response, with a wide variety of different contents concerning nutrition in cancer (e.g., dietary change, avoiding sugar/milk/meat/wheat, fasting, starving cancer cells, specific cancer diets, stress related to eating, etc.). The topics were then categorized into questions, patients’ direct contributions, and physicians’ indirect contributions.

A question was counted only if a member, who is registered in the forum, or a guest, who can post by just visiting a thread, sought help, or asked for advice on the evaluated nutrition topics and either initiated a thread with this question or asked in an already-persisting thread about a topic that concerned this thread.

The patients’ direct contributions contained all topics from all threads, which were categorized into six groups using a four-eye method.

For physicians’ indirect contributions, we assessed responses in which it was stated that the attending physician gave this recommendation to the member. They were divided into evidence-based and non-evidence based expert responses.

These findings were considered to be evidence-based when in line with the German S3 guidelines of Nutrition in Oncology [26], and non-evidence-based when not in line with the guidelines. Knowledge, to assess the content of each response and dividing it into different nutritional topics, was also gained from: fact sheets of the experts of the Working Group Prevention and Integrative Oncology of the German Cancer Society, available at (https://stiftung-perspektiven.de, last assessed 22nd of September 2024.)

## Results

### Quantitative assessment

In total, we assessed 2246 posts in 22 threads. The conversations lasted 3867 days from 24th of February 2013 to 27th of September 2023. The total duration of all conversations combined was 31,727 days, and the average duration of a thread lasted 1589 days.

The total number of visits to all threads was 654,100. Approximately 0,04 posts were made per day, and 12.37 visits per day were made in all 22 threads combined.

Fifteen threads (68%) were started by members of the forum, five threads were started by guests (23%), and two threads were started by members or guests that had been blocked- because they died (9%). All 22 threads were started by females, and the average age was 49.7 years. The majority of the patients who started the threads had breast cancer (*N* = 13; 59%), two had gynecological cancer (9%), one had gastric cancer (4.5%), one had anal cancer (4.5%), and the other five did not specify (23%). Overall, 13 patients stated in their profiles that they received chemotherapy, seven received adjuvant or neoadjuvant radiotherapy, four underwent additional operations, and nine did not specify which type of treatment they received. On an average, 452 posts were formulated by those individuals who began the discussions that we concentrated on, and their profiles were visited on an average of 2555 occasions.

### Content of threads concerning nutrition

Concerning all 22 threads that were assessed (Table 1), most posts were evaluated for nutrition for cancer, marking 42% of all threads. Interestingly, many studies have investigated the nutritional status during and after chemotherapy, weight loss, fasting, and an overall healthy diet. Most threads were started because patients were afraid to make mistakes and confusion about healthy nutrition that would be most beneficial for them. Specific cancer diets have also been discussed.

**Table 1:** Quantitative data of all threads concerning nutrition (*n* = 22)

Threads: Nutrition, Exercise and relaxation (from the topic)	Number of posts:	Number of visits:	Duration (d)	Posts per day:	Visits per day:
Nutrition for cancer	976	216,000	3378	0.29	63.94
Eating during chemotherapy	185	62,000	3148	0.06	19.70
Interaction between drug and nutrition	56	12,000	1140	0.05	10.53
What is now right or wrong	53	15,000	1494	0.04	10.04
Digestive side effects after chemotherapy	63	23,000	1603	0.04	14.35
Sugar and cancer	114	43,000	2711	0.04	15.86
MCT-diet	22	4800	421	0.05	11.40
Diet- vegan, vegetarian or ketogenic	83	24,000	1800	0.05	13.33
Fitness, nutrition and other changes	88	24,000	1472	0.06	16.30
Relationship between breast cancer and milk	66	14,000	1543	0.04	9.07
Recipes for healthy eating	111	41,000	3867	0.03	10.60
Recommendations against loss of appetite	27	5900	307	0.09	19.22
Ketogenic diet	19	7000	2268	0.01	3.09
What can I do during chemotherapy	72	42,000	1247	0.06	33.68
Fasting period	28	10,000	1915	0.01	5.22
Eating after the end of radiation/chemotherapy	19	13,000	1575	0.01	8.25
Vegan and vegetarian diet during chemotherapy (study)	24	9300	31	0.77	300.00
Soymilk from the supermarket	23	8100	4	5.75	2025.00
Phytoestrogens	34	14,000	1803	0.02	7.76
**Thread**: **complementary treatment options** (from the topic)					
Interactions with various herbs/foods, some cancer-promoting	103	22,000	3801	0.03	5.79
Fasting for healing - who has experience	64	13,000	1504	0.04	8.64
Fasting and chemotherapy	88	31,000	1705	0.05	18.18
**All Threads**:	**2246**	**654,100**	**31,727**	**0.04**	**12.37**

MCT-diet = Medium-Chain Triglyceride diet (Medium-Chain Triglycerides are fats that are made from e.g. coconut and palm kernel oils)

### Qualitative assessment

For the qualitative assessment, 5314 responses were obtained, resulting in a total of 41 topics. The total number of questions in the responses was 356, patients’ direct contributions were 4200 and indirect physician contributions were 758. Approximately 2.4 responses were counted in each post.

#### Content of questions from members

**Fig. 2:**
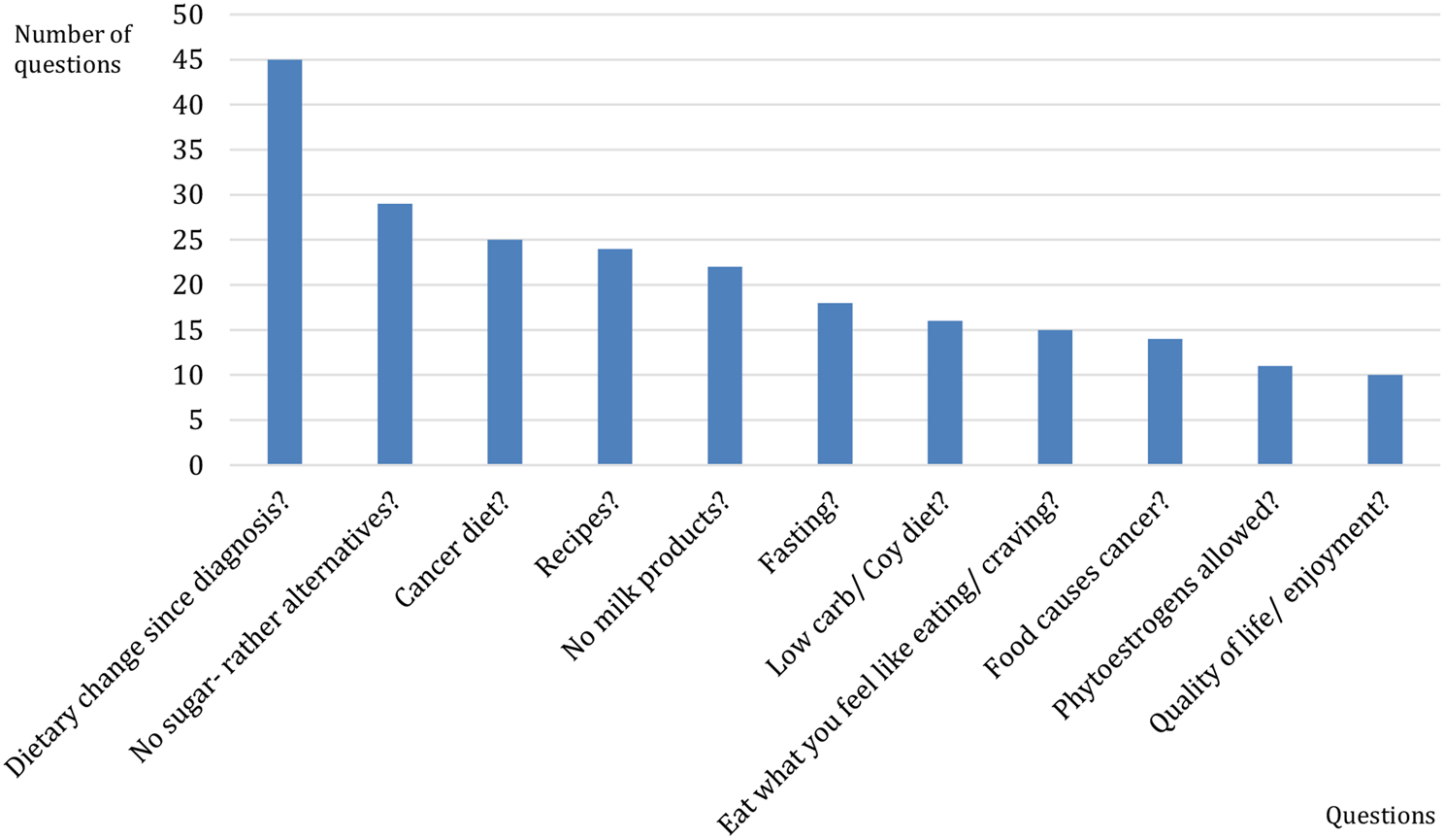
Commonest questions about nutrition among threads (Number of responses 5314)

Patients frequently sought information, asked for advice from other members, and valued the contribution of personal tips and ideas to their communication within the threads. These questions drove the initiation of most threads and the subsequent engagement of conversations and debates. The total number of questions in all responses was 356 for 41 topics, and commonest questions are illustrated in Fig. 2.

The most common question was asked 45 times to determine whether a dietary change had occurred since the diagnosis. An example of this is one member who started the thread “Nutrition for Cancer”, and she asked, “Have you changed your diet since the illness? One often hears and reads a lot about no carbohydrates, no sugar, no wheat flour, etc. How is it for you? I try to avoid sugar as much as possible, which is quite challenging for me, as I also enjoy snacking a lot. I would be very grateful for tips, advice, experiences, etc.”

Second, 29 members most commonly asked whether sugar was allowed or whether alternatives would be better. One member initiated a discussion on this topic by writing: “Now I’m starting to question whether sugar might be the potential cause of my cancer. I do not have any risk factors other than consuming sugar.”

Twenty-five members were asked about their cancer diets, 24 about their recipes, and 22 wanted to know if milk products should be avoided because of their beliefs about their carcinogenic effects, especially on hormonal breast cancer.

Additionally, 18 questions were asked about the participants’ experiences with fasting and recommendations for fasting. Much interest has also been shown in the low-carbohydrate diet/coy diet (16 questions). Additionally, 15 questions focused on whether it was acceptable to eat what one feels, such as eating and craving.

#### Patient direct contributions

We categorized topics that have been discussed most prominently in all responses and divided them into six groups: members’ emotions, problems of chemotherapy and radiotherapy influencing their diet, healthy diet and which dietary changes were made, and members´ goals.

**Fig. 3:**
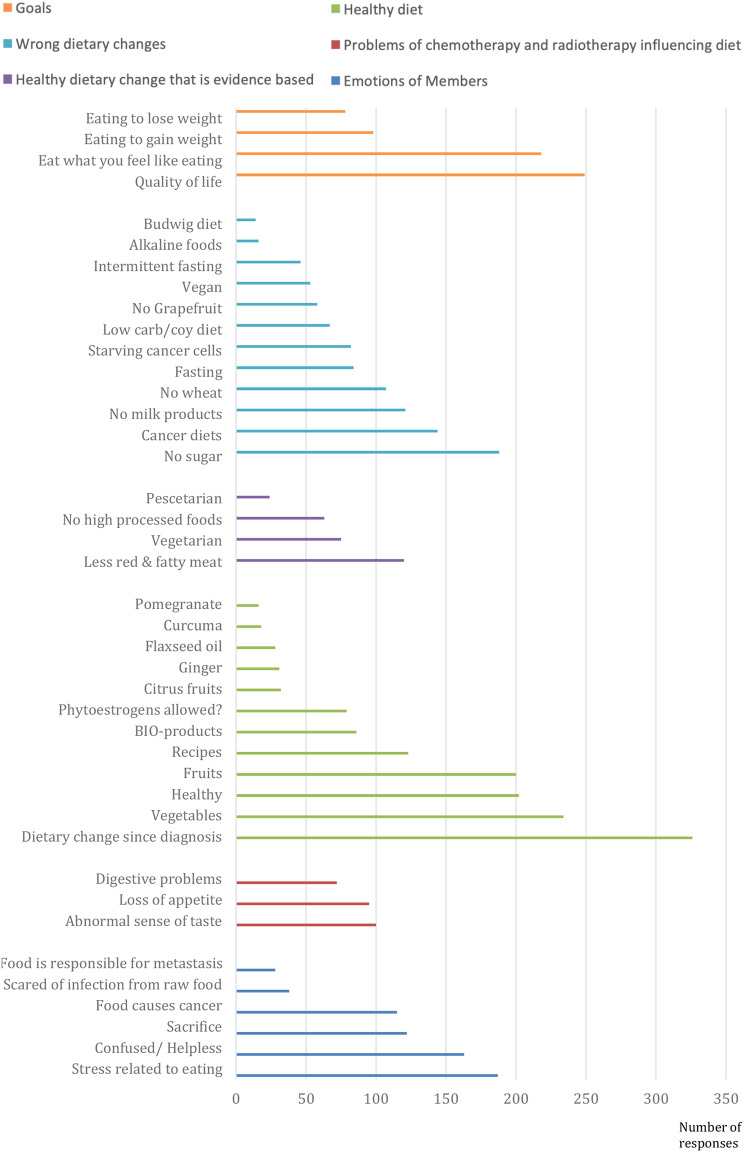
Topics of patient direct contributions (Number of responses 5314)

The total number of patient direct contributions is 4200, and all 41 topics are shown in Fig. 3, listing the most important to least important topics will demonstrate the significance of nutrition in cancer patients. For most members, “dietary change” after their diagnosis was very important, and most responses (326) focused on supporting and boosting their health and fighting against the disease. One member shared, “By changing my diet, I felt like I could do something against cancer myself, and that helped me cope with the illness.”

“Quality of life” had the second highest number of responses (249) and was categorized under.

goals that patients wanted to achieve through their diets. Most patients agreed that life should still be enjoyable, taste was important, and comfort in eating, and some reported that the results of their blood analyses improved when they ate what they craved. Similarly, topics such as “eat what you feel like eating” (218 responses) and “healthy” (202 responses) were important for members to support their well-being. Numerous comments also contained content with a negative weight; topics such as whether patients were permitted to “consume sugar” (188 responses)- led to discussions concerning the potential impact of sugar consumption on cancer growth and whether sugar was a contributing factor to their cancer. After such discussions, one member stated, “I always feel very sad reading such posts concerning sugar restriction. My friend consulted a nutritionist who advised complete sugar avoidance. This left her with no strength to continue the fight against cancer.”

Some members reflected a multitude of negative emotions, with “stress related to eating” being a topic in 187 responses. Additionally, a significant number of participants expressed feeling “confused and helpless” in 163 responses. One member shared, “Everyone recommends something different; it drives me insane. I don’t know what to do.”

Confusion was also stated by a member about initially becoming ill: “I lived so healthily and got cancer. Others who eat completely unhealthily remain healthy.”

The special “cancer diets” (144 responses) were discussed controversially. One example was a member who had regrets changing to such a diet: “I had completely avoided animal fats and dairy products after my metastasis diagnosis—I lost a lot of weight, and now I have no strength. In addition, the new metastases keep coming in waves.” Another example is a member who described “becoming increasingly uncertain as cancer diets vary so widely, from the Budwig Diet, to alkaline diet, to ketogenic diet; which one to choose?”

“Food causing cancer” (115 responses) was mentioned several times, and pressure to make a “sacrifice” (122 responses) connected to healthy nutrition was stated.

Additionally, 121 commented on “avoiding milk products” due to the belief that they cause cancer, similar to eating “less red and fatty meat” (120 responses). One member shared their belief that “milk being deemed hormone-laden due to milking practices of cows with calves; given her medical history of a hormone-dependent tumor, she eliminated dairy products on her diet.” Weight loss due to the disease and eating to compensate (98 responses) affected the nutrition of numerous members, as well as side effects from chemotherapy and radiotherapy, such as an “abnormal sense of taste” (100 responses) and “loss of appetite” (95 responses), which influenced their relationship to food. One member said, “I lost 25 kg within three months, looked like I had survived the worst famine, and was physically exhausted. This can’t be healthy either.”

Fasting” (84 responses) and “starving cancer cells” (83 responses) represent a big number of wrong dietary change and patients expressed the belief that fasting plays a significant role in the eradication of their cancer. One member stated, “I believe fasting is not further supported because the pharmaceutical industry does not profit from it; otherwise, there would be more recommendations concerning fasting.

The issue of whether a vegetarian (75 responses) or vegan diet (53 responses) would be advantageous was the subject of contentious debate in several fields.

Complete avoidance of “highly processed foods” (63 responses) has been reported.

Twenty-eight responses also focused on the viewpoint that “the growth of metastasis was caused by food.” A member expressed their concern, stating, “I fear that a cancer recurrence and growth of metastasis might be my responsibility, as I have not made sufficient dietary changes.”

#### Physician indirect contributions

Subsequently, we evaluated the responses to physicians’ indirect contributions and categorized them as either evidence-based or non-evidence-based expert responses. Physicians’ indirect contributions were considered valid only- if members indicated that they had received nutritional advice from qualified professionals, such as oncologists, nutritionists, or other medical experts, such as general practitioners, oncologists, or other specialists. The total number of patient indirect contributions was 758, which resulted in 558 evidence-based expert responses and 200 non-evidence-based expert responses.

**Fig. 4:**
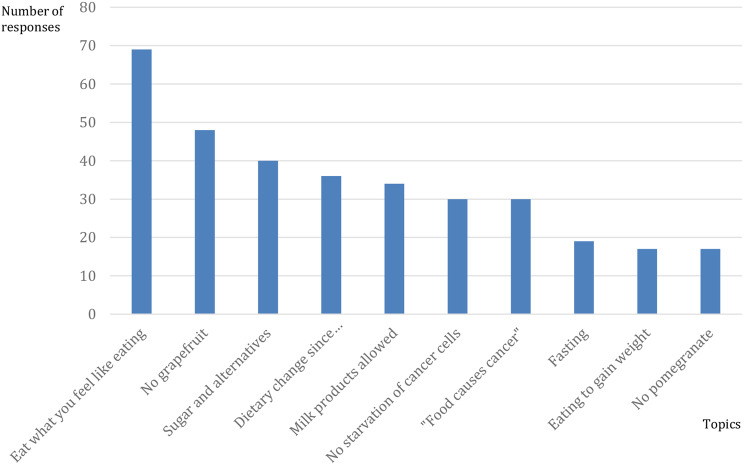
Most common topics from evidence-based expert responses (Number of responses 5314)

Figure 4 illustrates the evidence-based expert responses provided by patients. The members shared insights from their doctors and nutritional specialists, emphasizing the importance of eating based on personal preferences (69 responses), especially during chemotherapy when appetite loss and weight reduction significantly affected their health. An example of such a recommendation is a member’s statement, saying, “The doctors recommend a balanced diet, essentially what we have always been taught as healthy eating.” Another example of a member´s comment is “My oncologist advised against any specific diet; I should eat what I enjoy and tolerate.” 

Additionally, 48 members highlighted their oncologists’ strict recommendations to avoid grapefruit and pomegranate during therapy owing to potential interactions with chemotherapeutics. 

Most doctors advocated for an overall dietary change (36 responses) towards a healthy and balanced diet and explained these changes to patients. While reducing sugar intake was commonly suggested, it was clarified that complete avoidance was not necessary. One member advised, “My oncologist told me that there is no evidence proving that sugar promotes cancer growth. The Cancer Information Service (KID) also informed me in the same way.” Notably, 30 responses focused on dispelling the misconception that “starving cancer cells” would improve overall well-being, with members explaining that this approach does not impact cancer.

Furthermore, 30 responses addressed concerns about “food causing cancer,” with members correctly explaining this concept and bringing relief to many patients.

**Fig. 5:**
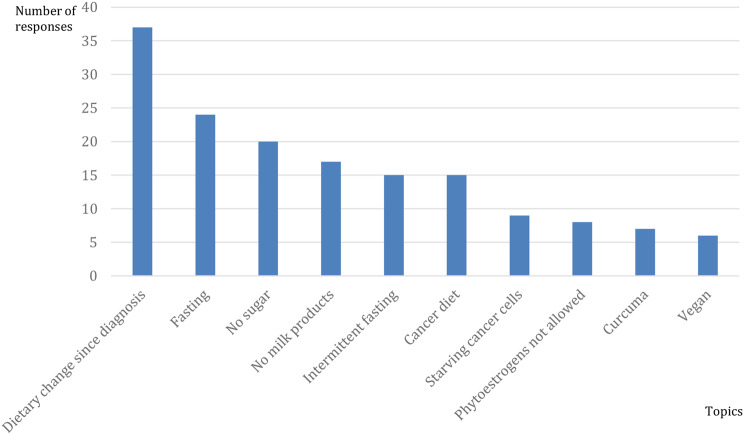
Most common topics from non-evidence-based expert responses (Number of responses 5314)

Conversely, the non-evidence-based expert responses are shown in Fig. 5.

There were instances of non-evidence-based advice regarding dietary changes- mentioned in 37 responses, which sparked debates and discussions within the forum.

One member stated, “At the beginning of my illness, the first oncologist suggested that I should adopt a low-carb diet and avoid sugar because cancer cells have a high affinity for sugar.”

Another commented, “My doctor recommended cutting out sugar, reducing carbohydrates, and practicing intermittent fasting, meaning not eating for approximately 13 hours. She mentioned that a ketogenic diet can contribute to improving the energy supply for patients and help preserve valuable muscle mass.”

Additionally, there were concerns regarding the inappropriate recommendation of fasting (24 responses) in certain situations. One member repeated that her doctor explained, “Certain diets and food regimens can counteract cancer. It is called ‘healing’ fasting and uniquely strengthens the immune system.”

Some members shared that their nutritionists, oncologists, or doctors advised against consuming sugar (20 responses), suggested avoiding dairy products (17 responses), and recommended cancer diets (15 responses) with a misguided intention of starving cancer cells (9 responses). One member shared, “My oncologist prohibited a lot of food for me two to three days before and after chemotherapy. No meat, no fish, no milk, no soy products, no heavily spiced foods, and especially no sugar.”

#### Threads during the Coronavirus pandemic

While analyzing the threads and qualitatively assessing the responses of each member of the forum of women’s self-help association against cancer, the corona-pandemic did not seem to have an impact on the nutrition of women with cancer. Two threads began after the beginning of the coronavirus pandemic. The total duration of the conversation during the COVID-19 pandemic in all threads lasted 1191 days, from the 30th of January 2020 until the 5th of May 2023. During this period, there was no obvious relationship between the pandemic and members activities.

## Discussion

Online forums are crucial for those with health issues to connect with and support each other.

Our study revealed that a self-help group forum may provide the basis for a detailed and intensive exchange on the topic nutrition for cancer patients. Members of the Women’s Self-help Association sought advice and help with healthy nutrition against cancer and wanted to educate themselves about nutrition for better prognostic outcomes of their disease. Information about diet and nutrition in patient-oriented and cancer-specific dietary recommendations is limited, as shown in our study, and it is important to understand that modifying or restricting diet can affect the prognosis of cancer patients.

Notably, from the beginning of the illness and treatment, many members reported undergoing changes in their dietary habits while looking for a strategy to support their fight against cancer. Some consciously chose a healthy diet, while others prioritized the intake of anything edible that felt manageable. Changes in diet may be helpful in case a patient turns to a healthier lifestyle [10]. However often patients formerly adhering to healthy nutrition after the diagnosis of cancer turn to a rigorous diet, such as vegan nutrition or even a “specific” cancer diet [17]. Patients diagnosed with cancer face heightened vulnerability to errors in nutritional intake, which may entail malnutrition and a serious loss in quality of life [8, 11].

Most advice was appreciated as beneficial and supporting coping mechanisms informing members of healthy nutrition, but some information led to uncertainty and distress.

Yet, we were able to identify several drawbacks of the information spread in the forum.

First, members sometimes share non-evidence-based information on the forum, which includes topics such as completely avoiding sugar, carbohydrates, or fasting, and in some cases, even starving cancer cells [15].

These recommendations may have a significant emotional impact and may even entail severe life-threatening undernutrition or malnutrition [20].

Analyzing discussions on cancer diets or whether vegan or vegetarian diets would be superior, it became obvious that some members were confused and helpless about what was allowed and what should be avoided.

Other threads point to the emotional contexts of nutrition and diet. Members shared their fear of having a negative impact on their prognosis when eating food, they enjoyed, and believed that it was their fault to disease with cancer. In contrast, some members correctly hinted that there should not be any unpleasant thoughts and concerns about their daily nutritional intake, coping with their illness and treatment is already distressing, and food should bring them joy. In fact, encouraging contributions to consume foods that bring you joy and satisfaction, reassuringly goes hand in hand with improved quality of life and evidence-based recommendations [11]; Tueros & Uriarte [22], 

There are some challenges for users of a forum; as evidence-based recommendations may depend on a concrete cancer situation, not all recommendations may be adequate for every patient. In fact, members reading evidence-based information must determine- whether the content applies to their individual situation and needs critical thinking and individual decision-making, when reading through posts, is mandatory, and both assume a high level of health literacy. It is good to be open-minded and learn about new strategies, but blindly trusting non-evidence-based recommendations can be rather dangerous, both mentally and physically [21].

Another difficulty for readers is- that the source of information does not always reflect the quality of information. As we have shown, information from qualified professionals, such as oncologists, nutritionists, or other medical experts, has not always been evidence-based and reliable. While we do not know, whether the miss-information was provided by the professional or whether the patient posting the information misunderstood the content, the importance of controlling the quality of the information exchange is high [5]. It is highly important for nutritional specialists and medical professionals to disseminate information that is evidence-based [9] and explain it in a language understandable to lay persons to avoid misunderstandings. (Turakulova [23]), 

Another important aspect is, that while contributions to the forum stem from individuals, and many posts convey individual experiences, those should be identified as such by other members. “Therefore, the request is not to patronize others but to allow everyone to find and follow their own path, engaging respectfully with one another. After all, we are not all scientists, but rather individuals who share our experiences here”, the previous comment from a member of the FSH forum perfectly illustrates why online self-help forum may be helpful, when information and recommendations are reconsidered carefully, as not every advice is suitable for each and everyone’s needs [18].

A possible solution for these problems is a method of improving reliability of posts in a forum, establishing a group of moderators who check all new posts, and remove or comment on non-evidence-based or even false posts. If these moderators are members of the forum and laypersons, they might profit from a training and an advisory council of experts, as they already perform it on the FSH website [24].

A well-solved topic in the forum is the discussion whether vegan or vegetarian diets would be superior. Concerning veganism, evidence-based recommendations and results were expressed in the threads, indicating that a vegetarian diet may not be superior to a diet that includes meat and fish; however, vegan diets may lead to nutritional deficiencies.

Considering the widespread usage of social media by patients, one patient understanding an item may multiply this evidence-based recommendation from a physician, for example, to members of a forum- who are also looking for proper (nutritional) guidance [16].

On the other hand, highly engaged users with many contributions may also become a danger if they adhere to a non-evidence-based or even extreme theory.

To increase the quality of information in forums, a collaboration between experts contributing evidence directly or indirectly and members contributing experiences and reassuring other patients may help to spread high-quality information on nutrition and reassure patients to adhere to this evidence [14].

This may make discussions more trustworthy and the content in the threads more reliable.

Additionally, similar forums as the EuroCancerComs project that allowed participants to engage in live activities, discussions, and examinations related to nutrition and cancer care should be further supported for cancer patients, similar forums as the Women´s Self-help Association Against Cancer [12]. This project is a proposed solution for informational exchange in forums, suggesting that live activities can also be held in the forums and that the forums should continue to be supported [25], because the greater the reach and the more exchange of accurate information, the better.

This study highlights the potential of online forums to enhance the training of doctors, particularly in addressing cancer-related nutrition. Forums offer valuable insights into patient concerns, misconceptions, and emotional responses, which can be used in medical education to improve communication and guidance skills. Training could focus on recognizing misinformation and providing clear, evidence-based advice in an accessible manner. Incorporating case studies from forums into medical programs would help doctors to better address patients’ nutritional challenges and support informed, individualized decision-making.

### Strengths and contribution to the literature

This study provides valuable insights into cancer-related nutrition and the role of online forums in addressing cancer patients’ concerns regarding nutrition. By analyzing 2,246 posts over a decade, with 5,314 responses, it highlights the sustained relevance of forums for patient engagement. The comprehensive qualitative assessment of 41 topics enabled a deep understanding of patients’ emotions, thoughts, and challenges, helping to identify critical gaps in healthcare and the impact of misinformation, particularly on vulnerable and desperate individuals. Evaluating each comment and its context allowed us to empathize with patients, understand their struggles, and recognize how misinformation can exacerbate distress. The findings underscore the importance of promoting evidence-based discussions, improving health literacy, and incorporating these insights into medical training to better address patient needs.

### Limitations

There are three limitations in our study. First of all, we only assessed one forum, which may not be representative for other forums of cancer patients with other types of cancer, prevailing male instead of female patients or patients living in other countries.

Second of all, some members are more active than others; and while there are occasions where we could acknowledge these members, it is not always possible to consistently identify them. This is important because a potential oversight of their repetition of opinions could not be excluded, and in case of their high activity in the threads, they could have a dangerous influence when non-evidence-based information is shared.

Moreover, we only analyzed a subgroup of threads (topics: “side-effect Management”, “coping with diseased-relatives”, “how can I deal with my anxious thoughts”, “and more”, “nutrition, exercise and relaxation” and “complementary treatment options”) from 6th of January 2013 until 27th of September 2023. Nutrition might also be addressed in other threads and other contexts which might have produced additional aspects.

## Conclusion

This study highlights the importance of addressing myths and incorrect beliefs about cancer-related diets, emphasizing the need to spread evidence-based knowledge. A well-managed online forum can serve as a crucial source of support for cancer patients, offering both accurate nutritional information and reassurance in moments of uncertainty. These platforms enable members to share personal experiences, coping strategies, and practical insights, fostering a sense of community and mutual encouragement during challenging times.

Forums also play a vital role in disseminating practical, evidence-based information, empowering patients to make informed decisions about their health. Beyond providing mental support, they help keep members updated on the latest developments in treatments, nutrition, and self-care. By offering a judgment-free space for individuals to express their fears, frustrations, and successes, forums can significantly enhance emotional well-being and resilience.

For experts and societies aiming to provide high-quality information, collaborating with forums could complement traditional patient education tools such as books, leaflets, and websites. However, it is critical that self-help organizations ensure the evidence-base of the information shared while maintaining independence and prioritizing the member-to-member interaction that makes forums unique.

Future research should focus on how forums can better integrate expert advice, while maintaining their supportive environment, and explore the long-term benefits for healthcare systems. It should also examine the impact of evidence-based nutrition guidance, gaps in patient-physician communication, and how forum dynamics influence dietary behavior. Additionally, research could look into cultural and demographic factors affecting nutritional beliefs and assess the safety of popular diets. By addressing misinformation and strengthening these platforms, forums can become a more powerful resource, providing cancer patients with both valuable information, and emotional support.

## Data Availability

No datasets were generated or analysed during the current study.
